# Conflicts of Interest: Blumberg’s Response

**Published:** 2004-10

**Authors:** Bruce Blumberg

**Affiliations:** Department of Developmental and Cell Biology University of California, Irvine Irvine, California, E-mail: blumberg@uci.edu

In his letter, Goozner suggested that I may have failed to disclose a competing financial interest in regard to an article published in *EHP*. In this article, “Highly Chlorinated PCBs Inhibit the Human Xenobiotic Response Mediated by the Steroid and Xenobiotic Receptor (SXR)” (Tabb et al. 2004), we described differences in how humans and rodents respond to highly chlorinated polychlorinated biphenyls (PCBs). We concluded that rodents may not be appropriate models for exposure to the class of PCBs discussed in the article and suggested that previous research using rodent models to predict the effects of these PCBs on humans may need to be re-evaluated in light of our findings.

In his letter, Goozner noted that I am the co-inventor of U.S. Patent 6,391,847 (“Method, polypeptides, nucleotide sequence of XOR-6, a vitamin D-like receptor from Xenopus”). This patent describes a frog nuclear receptor, now referred to as the benzoate “X” receptor (BXR). As I pointed out on 24 June 2004 in an e-mail message to Goozner, I am not the owner of this patent; the patent is owned and controlled by the Salk Institute for Biological Studies, where I was employed from 1992 to 1998.

It is difficult to understand how Goozner reasons that the frog BXR patent is related in any way to our article on rodent and human SXR (Tabb et al. 2004). My laboratory (Grün et al. 2002) and another laboratory (Moore et al. 2002) have shown that BXR and SXR (also known as PXR) are functionally distinct and that BXRs do not function as xenobiotic receptors. Therefore, there is no functional link between BXR and SXR/PXR, as I also pointed out to Goozner in my e-mail.

The *EHP* Instructions to Authors (EHP 2003) defines a competing financial interest thusly: “Competing financial interests may include, but are not limited to, grant support, employment (recent, present, or anticipated), and personal financial interests by the authors, immediate family members, or institutional affiliations that may gain or lose financially through publication.” Therefore, for a competing financial interest to exist, there must be at least some realistic probability at the time of submission that publication of the article in *EHP* would lead to financial gain or loss to the authors, their immediate family members, or institutional affiliations. Considering that there is no functional similarity between frog BXR and rodent and human SXR, it is not reasonable to infer that publication of the article regarding the function of SXR in rodents and humans (Tabb et al. 2004) would have any influence on financial interests related to U.S. Patent 6,391,847. Therefore, no potential competing financial interest existed at the time of submission or publication of this manuscript, and as a result, none was disclosed.

In view of these ongoing discussions of interpreting and perhaps heightening the standards regarding disclosure, I wish to inform you of a patent that I just learned was recently issued: U.S. Patent 6,756,491, “Steroid-activated nuclear receptors and uses therefore” was issued on 29 June 2004, over 4 months after the publication of our article (Tabb et al. 2004) in *EHP*. I am the co-inventor of this patent, which teaches the sequence of SXR and its nucleotide response elements. Because this patent is owned and controlled by the Salk Institute, I was unaware of its status. Had this patent been issued at the time of submission or publication of the article (Tabb et al. 2004) (or had I known that it would issue shortly), I would have disclosed it as a potential competing financial interest. In contrast to the BXR patent, this patent meets the tests described above. It is functionally connected to the subject matter of the article, it clearly has commercial value, and it is foreseeable that I will receive some fraction of whatever income the Salk Institute receives in the course of licensing it to interested parties. Whether or not publication of the article in *EHP* will lead to a financial gain or loss as required by *EHP* policy remains to be seen.

I fully support *EHP’*s competing financial interest policy. Goozner argues in his letter to *EHP,* and in e-mails to me, for a relatively extreme interpretation of what constitutes a competing financial interest, which, as far as I understand it, is beyond the scope of the current *EHP* policy. Whether such an interpretation will become the norm for the scientific community is a matter for future discussion. Although scientists make a good faith effort to comply with disclosure clauses, most are not well trained in understanding the legal nuances involved. It would be very helpful if policies ultimately adopted by journal editorial boards were clearly stated and included appropriate examples so that authors can readily understand the requirements and more effectively comply with the policy.
